# Dietary Habits and Nutritional Status Among Polish Police Officers: A Cross-Sectional Study Within the National Health Programme

**DOI:** 10.3390/nu18142385

**Published:** 2026-07-22

**Authors:** Anna Anyżewska, Roman Łakomy, Tomasz Lepionka, Andrzej Tomczak, Jerzy Bertrandt

**Affiliations:** 1 School of Health and Medical Sciences, VIZJA University, Okopowa 59, 01-043 Warsaw, Poland; 2 Independent Researcher, 05-506 Lesznowola, Poland; 3 Laboratory of Next Generation Sequencing, Biological Threats Identification and Countermeasure Centre, Military Institute of Hygiene and Epidemiology, Lubelska 4, 24-100 Pulawy, Poland; tomasz.lepionka@wihe.pl; 4 WSB Merito University in Poznan, 61-895 Poznan, Poland; 5 Faculty of Economic Sciences, John Paul II University in Biala Podlaska, Sidorska 95/97, 21-500 Biala Podlaska, Poland

**Keywords:** dietary behaviours, food frequency questionnaire, adiposity, body composition, body mass index, fat mass index, uniformed services, occupational health

## Abstract

**Background/Objectives**: Police is an occupational group characterised by high and variable physical demands, shift work, and the need for sustained operational readiness. Despite the importance of maintaining good health in uniformed services, relatively little is known about dietary habits and nutritional status among police populations, particularly in Poland. Therefore, the aim of this study was to characterise dietary habits and nutritional status among Polish police officers participating in the National Health Programme. **Methods**: This cross-sectional study included 262 Polish police officers (216 men and 46 women) aged 19–64 years. A 61-item food frequency questionnaire (FFQ) was used to assess dietary behaviours and bioelectrical impedance analysis was used to assess body composition. **Results**: Suboptimal food consumption frequency patterns were observed for several food groups, including fruits, vegetables, whole grains, dairy products, nuts, and seeds. Female police officers reported more frequent consumption of fruits, vegetables, and grains, and less frequent consumption of processed meats, animal fats, sugar-sweetened beverages, energy drinks, beer, and spirits than male officers. Normal body weight according to BMI criteria was observed in 33% of participants, whereas only 16% of participants had normal fat mass index values. Excessive body weight was observed in 67% of participants, and excess fat in 82% of participants. **Conclusions**: The study provides a comprehensive characterisation of dietary habits and nutritional status among Polish police officers within the National Health Programme. These findings suggest the need for nutritional education, health promotion activities, and continued monitoring of dietary habits and nutritional status in this occupational group.

## 1. Introduction

Police work is a public service focused on protecting life, health, and property; maintaining public safety and public order; and detecting crime [1]. Since police duties require constant readiness to respond to unpredictable operational demands, officers must maintain not only appropriate physical and psychological capacities but also good overall health. Adequate nutrition represents a key modifiable determinant of health, functional capacity, and operational readiness in this occupational group. Police tasks include, among others, emergency response, suspect pursuit, crowd control, and the use of direct coercive measures. In addition, irregular working hours, weekend and holiday shifts, night patrols, and shift work may adversely affect multiple dimensions of health and well-being. These occupational characteristics may contribute to unfavourable lifestyle behaviours, including irregular eating patterns and suboptimal diet quality.

Health-related behaviours among uniformed service personnel have received increased scientific attention in recent years. Police officers represent a distinct occupational group exposed to specific health challenges associated with demanding service conditions, including shift work, occupational stress, and varying operational demands [2,3]. Previous studies in police and other uniformed service populations have shown that energy expenditure and lifestyle behaviours may vary substantially across different service contexts, depending on duty type, occupational role, sex, and rank [4,5,6,7,8,9]. Shift work may additionally influence dietary behaviours and nutritional status. A recent study among police officers on patrol indicated that participants consumed more calories on rest days than evening- and night-shift days, which was associated with higher fat and carbohydrate intake [10]. Another study from the UK reported significantly poorer diet quality during all shift types compared with rest days [11]. Circadian disruption associated with shift work has also been linked to adverse metabolic outcomes that may interact with dietary behaviours and nutritional status [12].

Despite the importance of maintaining good health in uniformed services, relatively little is known about dietary habits and nutritional status in police populations, particularly in Poland. Previous studies conducted among uniformed service personnel have identified several dietary inadequacies, including insufficient consumption of fruits and vegetables, cereal products, dairy products, nuts, and seeds among soldiers [13] and border guard officers [14]. Abnormalities in nutritional status, such as elevated BMI and increased body fat percentage, have also been reported [13,14,15,16]. However, comparable data regarding Polish police officers remain limited.

The present study was conducted within the framework of the National Health Programme, under which dietary habits and nutritional status were assessed in several Polish uniformed service populations. Previous analyses from this programme described dietary habits and nutritional status among soldiers [13] and border guard officers [14]. The inclusion of police officers extends this line of research and enables direct comparisons across major Polish uniformed services using a consistent methodological approach. Police officers represent another important occupational group exposed to shift work, occupational stress, and varying operational demands that may influence lifestyle behaviours, including dietary habits. Unlike previous studies conducted in isolated occupational settings, the present study applies a consistent methodological framework used across several Polish uniformed service populations, enabling direct comparisons between police officers, soldiers, and border guard officers. Although several studies have examined selected health behaviours among police officers, comprehensive data combining detailed dietary assessment with objective measures of nutritional status remain limited, particularly in Polish police populations. Therefore, the aim of this study was to characterise dietary habits and nutritional status among Polish police officers participating in the National Health Programme. Exploratory analyses of relationships between dietary behaviours and selected nutritional status indicators were additionally performed and should be interpreted as secondary findings rather than the primary focus of the study.

## 2. Materials and Methods

This cross-sectional observational study was conducted among Polish police officers within the framework of the National Health Programme 2016–2020. The primary objective was to assess food consumption frequency patterns and nutritional status, including body composition, and to explore associations between dietary behaviours and selected nutritional status indicators. Data were collected using a food frequency questionnaire, anthropometric measurements, and bioelectrical impedance analysis. A total of 262 police officers (216 men and 46 women) from Poland participated in the study. Participants were recruited from different regions and police units across Poland, including police schools and police training centres that voluntarily participated in the programme. The study sample included police officers representing different service settings across the country. Informed consent was obtained from all participants. The study was conducted in accordance with the Declaration of Helsinki and approved by the Ethics Committee of the Military Institute of Hygiene and Epidemiology (1/XXI/2016).

### 2.1. Food Consumption Frequency Assessment

A 61-item food frequency questionnaire (FFQ), validated for the Polish population [17], was used to assess dietary behaviours. The questionnaire was slightly adapted by adding two additional response categories (“quarterly or less often” and “once a week”), consistent with previous studies conducted in Polish uniformed service populations [13,14]. These modifications were introduced to improve the precision of dietary frequency assessment while maintaining comparability with earlier investigations conducted within the National Health Programme. Police officers were asked to indicate how often they had consumed 61 food products in the past 12 months. For each product, participants could choose one of eight frequency categories. Reported frequencies were converted to daily frequency values (times/day) as follows: never or almost never—0.003 (1/365), once a quarter or less often—0.01 (4/365), once a month or less often—0.03 (1/30), a few times a month—0.08 (2.5/30), once a week—0.14 (1/7), several times a week—0.57 (4/7), every day—1, several times a day—2. Converted frequencies were also summed within 11 main food groups identified as: 1. Fruits, vegetables, and potatoes; 2. Seeds of legumes; 3. Cereal products; 4. Dairy products and eggs; 5. Meat products; 6. Fish; 7. Fats; 8. Nuts and grains; 9. Sweets and snacks; 10. Soft drinks; 11. Alcoholic beverages. The questionnaire assessed the frequency of consumption of selected food products and did not provide information on portion size, total food intake, energy intake, or nutrient intake.

### 2.2. Nutritional Status Assessment

Body mass and body composition (fat mass and visceral fat level) were assessed using the TANITA MC-780 multi-frequency bioelectrical impedance analyser (Tanita Corporation, Tokyo, Japan). Height was measured using the TANITA HR-001 stadiometer (Tanita Corporation, Tokyo, Japan), with participants’ heads positioned in the Frankfurt horizontal plane [18]. Participants received standardised instructions on preparation for the measurements and were assessed barefoot using the standard 8-polar hand-to-foot mode. Pregnancy and the presence of a cardiac pacemaker constituted exclusion criteria for the body composition assessment. Body weight was recorded to the nearest 0.1 kg using the integrated electronic scale (measurement range: 0–270 kg). During the measurement, participants held the hand electrodes with their arms slightly abducted to avoid contact with the trunk, while the inner thighs remained separated. Participants were instructed to avoid vigorous physical activity before the assessment. Measurements were performed around midday, at least three hours after a meal, in accordance with the manufacturer’s instructions, with all metal objects (e.g., jewellery and keys) removed before the assessment Two additional indicators, body mass index (BMI) and fat mass index (FMI), were calculated using the following formulas: BMI = body weight/height^2^ [kg/m^2^], FMI = fat mass/height^2^ [kg/m^2^]. BMI classification scales from the World Health Organisation (WHO) [19] and FMI classification scales described by Kelly et al. [20] were used.

### 2.3. Statistical Analysis

The PS IMAGO PRO 11.0 (IBM SPSS Statistics 31, Armonk, NY, USA) programme was used for all statistical analyses. The Shapiro–Wilk test was used to verify the normality of variable distribution. Due to the non-normal distribution of the analysed variables, the Mann–Whitney U test was used to compare food consumption frequency between males and females. For comparisons performed using the Mann–Whitney U test, effect sizes (r) were calculated by dividing the standardised test statistic by the square root of the total sample size. These comparisons constituted the primary descriptive analyses of the study. Spearman’s correlation analysis was additionally conducted as an exploratory analysis to assess the potential associations between food consumption frequency and four nutritional status indicators (BMI, FMI, body fat percentage—FAT%, and visceral fat level—V FAT L). These analyses represented secondary analyses and did not constitute the primary focus of the study. Due to the exploratory character of the analyses and multiple statistical comparisons, the Benjamini–Hochberg false discovery rate (FDR) correction was applied separately within two predefined families of tests. For sex-related comparisons corrections were performed independently for major food groups (11), and for individual food products (61 items). For exploratory correlations analyses, FDR correction was applied separately for each nutritional status indicator (BMI, FMI, FAT%, and V FAT L) stratified by sex, with independent corrections for major food groups and individual food products. Statistical significance was set at *p* < 0.05. To account for multiple comparisons, *p*-values were adjusted using the false discovery rate (FDR) procedure. *p*-values remaining statistically significant after FDR correction are shown in bold in the corresponding tables.

## 3. Results

### 3.1. Characteristics of the Study Population

The study included 262 police officers from Poland, aged 19–64 years, of whom 82% were men. Regarding place of residence, 31% of respondents lived in rural areas, 34% in towns, and 36% in cities with more than 100,000 inhabitants.

### 3.2. Food Consumption Frequency

Many suboptimal dietary behaviours were observed in the studied group of Polish police officers, particularly low consumption frequencies of fruits, vegetables, fish, dairy products, nuts, and grains (Table 1). Nearly half of the participants (49%) reported consuming fruits several times per week, whereas only 29% declared daily fruit intake, including only 6% consuming fruits more than once per day. A comparable pattern was observed for vegetables: 34% of participants reported daily consumption (7% several times per day), while 43% consumed vegetables several times per week. Wholegrain products (so-called dark bread) were consumed daily by 31% of participants, compared with 29% for refined (white) bread. Dairy products were generally consumed less than once per day by most participants. Meat products, particularly processed meats (e.g., cold cuts and sausages), were consumed more frequently than fish. Approximately three-quarters of participants consumed both lean and fatty fish less than once per week (76% and 73%, respectively). Nuts and grains were consumed infrequently, with only 22% and 18% of participants, respectively, consuming them more than once per week. Participants consumed sweets more frequently than salty snacks. Energy drinks were never or almost never consumed by 41% of participants, while 19% consumed sugar-sweetened beverages more than once per week.

### 3.3. Sex Differences in Dietary Habits

Statistically significant differences between male and female officers were identified for three of the 11 analysed food groups (Table 2). Women reported higher consumption frequency of fruits, vegetables, and potatoes compared with men (7.44 vs. 5.33; *p* < 0.001), whereas men reported higher consumption frequency of non-alcoholic beverages (0.76 vs. 0.57; *p* = 0.001) and alcoholic beverages (0.37 vs. 0.14; *p* < 0.001). Overall, women more frequently consumed fruit- and vegetable-based products, whereas men reported higher consumption of processed meat products, animal fats, added sugars, sugar-sweetened beverages, and alcoholic drinks.

### 3.4. Nutritional Status

Normal body weight according to BMI criteria was observed in 33% of participants (Table 3). Normal fat mass index (FMI) values were identified in only 16% of participants, whereas normal visceral fat levels (V FAT L) were observed in the majority of the study group (95%). These findings indicate substantial differences in the classification of nutritional status depending on the indicator applied.

### 3.5. Exploratory Associations Between Dietary Habits and Nutritional Status Indicators

Exploratory analyses identified several associations between food consumption frequency and nutritional status indicators; however, these associations were heterogeneous and differed by sex (Table S1 in the Supplementary Materials). Due to multiple testing, the Benjamini–Hochberg false discovery rate correction was applied separately to sex comparisons in food consumption frequency and to exploratory correlation analyses (Table S2 in the Supplementary Materials). After FDR correction, female officers still reported higher consumption frequencies of fruits and vegetables, including apples, pears, green leafy vegetables, tomatoes, cucumbers, squash, zucchini, pumpkin, and root vegetables. Women also consumed sweetened milk products and grains more frequently, whereas male officers reported higher consumption frequencies of processed meat products, animal fats, added sugar, sugar-sweetened beverages, energy drinks, beer, and spirits. Among the exploratory correlation analyses, only a weak positive association between the frequency of vodka and spirits consumption and body fat percentage among male officers remained statistically significant after FDR correction (Rho = 0.230, adjusted *p* = 0.001). All other associations should be interpreted cautiously.

## 4. Discussion

The present study provides detailed data on dietary habits and nutritional status among the studied group of Polish police officers, a population that remains insufficiently characterised in the literature. Several dietary inadequacies were identified, including low consumption of fruits, vegetables, nuts, seeds, dairy products, and whole grains, as well as fish, alongside relatively frequent consumption of processed meat products. These findings are consistent with previous studies conducted among Polish soldiers and border guard officers participating in the National Health Programme [13,14]. From a public health perspective, these findings indicate suboptimal adherence to international dietary guidelines. The World Health Organisation recommends a minimum intake of 400 g of fruits and vegetables per day to reduce the risk of non-communicable diseases [21]. Although the present study assessed consumption frequency, the observed patterns suggest that fruits and vegetables were not consumed daily by most participants. In addition, dietary guidelines emphasise regular consumption of nuts and seeds as part of cardioprotective dietary patterns [22], such as the Mediterranean diet [23] or the healthy planetary diet [24]. Particular attention should be given to the insufficient consumption frequency of key food groups with well-established health benefits. Fruits and vegetables are major sources of dietary fibre, vitamins, minerals, and bioactive compounds, whereas dairy products contribute substantially to calcium, and high-quality protein intake. Nuts and seeds provide unsaturated fatty acids, plant protein, fibre, and antioxidant compounds. The observed dietary pattern was characterised by relatively infrequent consumption of several nutrient-dense food groups and relatively frequent consumption of processed meat products, which may warrant further attention in health promotion programmes targeting police officers.

A further concern is the imbalance between the relatively frequent consumption of processed meat products and low frequency fish consumption. Previous studies have linked frequent consumption of processed meats to increased risk of cardiovascular disease, type 2 diabetes, and certain cancers [25], whereas regular fish consumption, particularly fatty fish, has been recognised as an important component of cardioprotective dietary patterns [26]. Although the present study assessed only consumption frequency, the observed imbalance between fish and processed meat may be important from a public health perspective. This imbalance is particularly relevant in occupational groups exposed to elevated stress and irregular work patterns, where baseline cardiometabolic risk may already be increased. Similar dietary imbalances have been reported in other uniformed service populations, further supporting the need for targeted nutritional interventions in these groups. Given the cross-sectional design and frequency-based dietary assessment, the present findings should be interpreted as descriptive and associative rather than causal.

Previous studies conducted in tactical and uniformed populations suggest that occupational conditions may influence dietary behaviours and meal patterns. Similar dietary inadequacies have previously been observed among Polish soldiers and border guard officers participating in the National Health Programme [13,14]. Nutritional analyses in German riot police demonstrated insufficient provision of several recommended food groups, including whole grains, fish, nuts, and legumes [27]. In addition, the results of other study showed that higher caloric and protein intake was associated with poorer psychomotor vigilance performance during day shifts [28]. A systematic review of tactical personnel likewise reported that fat intake frequently exceeded recommended values, whereas carbohydrate and total energy intake were often insufficient [29]. Taken together, these findings indicate that dietary behaviours observed in police and other tactical occupations occur within specific occupational contexts characterised by irregular schedules, shift work, and limited opportunities for regular meal consumption and food choices during duty.

The observed food consumption patterns should be interpreted in the context of both occupational and population-level determinants. Recent national reports indicate that dietary habits in the adult Polish population remain suboptimal, including insufficient consumption of fruits, vegetables, wholegrain cereal products, milk and dairy products, fish, and nuts, accompanied by frequent consumption of red and processed meat products [30,31,32]. Similar dietary inadequacies were observed among the studied police officers. Sex-related differences observed in the present study are also consistent with findings reported for the general Polish population. National surveys have shown that men tend to consume fruits and vegetables less frequently and consume red and processed meat products more frequently than women [30,33]. Comparable sex-related differences have also been reported among police personnel participating in the Airwave Health Monitoring Study [34]. Taken together, these findings suggest that dietary behaviours observed among police officers should be interpreted within the broader context of dietary patterns in the adult Polish population, while occupational characteristics discussed in previously studies may also contribute to shaping food-related behaviours in uniformed services.

Exploratory analyses identified several associations between food consumption frequency and nutritional status indicators. However, these findings should be interpreted cautiously because the analyses included multiple statistical comparisons, most associations were weak, and the majority did not remain statistically significant after FDR correction. After adjusted for multiple testing, only a weak positive correlation between the frequency of vodka and spirits consumption and FAT% among male police officers remained statistically significant. All other observed associations should be regarded as exploratory analyses. Interpretation of these associations is additionally complicated by the multifactorial aetiology of obesity and body composition changes, which are influenced not only by dietary behaviours but also by physical activity, sleep quality, smoking, alcohol consumption, shift work characteristics, hormonal factors, and other lifestyle-related variables [35,36,37,38]. Comparable patterns have been observed in general adult populations, in which associations between diet and obesity are often attenuated after adjustment for lifestyle-related and socioeconomic confounders [7,34,39,40,41]. Accordingly, the present findings should be interpreted within the broader context of the multifactorial aetiology of obesity, in which frequency-based dietary measures alone may not fully capture the determinants of body composition in this occupational group and other structured populations.

The high prevalence of excess body weight and adiposity observed in this study is consistent with findings reported in other Polish uniformed service populations examined within the National Health Programme. Previous analyses conducted among soldiers and border guard officers also demonstrated a high prevalence of excess body weight according to BMI criteria [13,14]. Similar concerns have been reported internationally among military personnel, police officers, and other tactical occupations [35,42,43,44]. In the present study, 67% of police officers were classified as overweight or obese according to BMI criteria, a proportion comparable to that previously observed among Polish border guard officers and higher than reported among soldiers participating in the National Health Programme [13,14]. According to the World Health Organisation, in 2022 approximately one in eight people worldwide was living with obesity; in addition, 2.5 billion adults aged 18 years and older were overweight, including 890 million living with obesity [45]. Similar findings have been reported in police and military populations from other countries, indicating that excess body weight remains a common challenge in uniformed services [35,36,42,44,45]. Wide variability in BMI values has also been reported in previous studies in police populations [10,15,46,47]. At the same time, the present results support the interpretation that BMI alone may be insufficient for assessing nutritional status in physically active adults. While 33% of participants had normal BMI values, only 16% had normal FMI values. These findings highlight the importance of complementing anthropometric indicators with body composition assessment and are consistent with previous observations in police officers and other uniformed service populations [13,14,15,16,48,49].

The discrepancy between the proportion of participants with normal BMI values (33%) and with normal FMI values (16%) warrants further consideration. These findings indicate that different indicators may provide substantially different assessments of nutritional status in physically active occupational groups. In addition, the high proportion of participants with normal visceral fat levels despite the high prevalence of excess adiposity according to FMI highlights the complexity of nutritional status assessment. These indicators reflect distinct components of body composition, with FMI representing total adiposity and visceral fat level reflecting central fat distribution, which is more strongly associated with increased cardiometabolic risk and insulin resistance [50,51]. Therefore, elevated FMI or total body fat percentage may coexist with normal visceral fat levels, particularly in physically active adults [14,52]. Previous studies indicate that higher levels of physical activity are associated with lower total and visceral adiposity [53]. However, research conducted in physically active adults has shown that the relationship between total and visceral adiposity may remain relatively weak below approximately 20% body fat, suggesting that visceral fat accumulation does not necessarily increase proportionally with overall adiposity [52]. These findings also highlight broader challenges in the assessment and classification of obesity, as different anthropometric and body composition indicators may lead to divergent interpretations of nutritional status [54]. In recent years, increasing attention has been given to phenotypes such as normal-weight obesity (NWO) and metabolically obese normal-weight (MONW), which illustrate that traditional or single-indicator approaches may not adequately reflect metabolic risk [55,56]. Although these phenotypes were not directly assessed in the present study, they provide a relevant conceptual framework for interpreting discrepancies between body composition indicators. Similar observations have also been reported in other uniformed service populations examined within the National Health Programme, supporting the need for a multidimensional approach to nutritional status assessment in physically active occupational groups [14]. From a practical perspective, the findings may support the development of occupational health strategies aimed at improving dietary behaviours and nutritional awareness among police officers. However, the present findings should be interpreted within the limitations of the cross-sectional study design and the frequency-based, self-reported dietary assessment.

## 5. Limitations

This study has several limitations. Due to its cross-sectional design, causal inferences cannot be established. Dietary data were collected using a self-reported FFQ with a 12-month recall period, which may introduce recall and reporting bias. The assessment was based on consumption frequency only and did not include portion sizes or total energy intake. In addition, the FFQ was slightly modified by introducing two additional response categories, which may have affected the comparability and measurement properties of the original validated instrument. The statistical analyses were exploratory and included multiple comparisons, which increase the possibility of type I error. Therefore, statistically significant associations should be interpreted cautiously. Another limitation is the absence of analyses of confounders related to lifestyle characteristics. The study sample was voluntary and predominantly male, reflecting the occupational structure of the Polish police force, which limited sex-specific analyses and may affect the generalisability of the findings. Additionally, potential confounding factors such as physical activity, sleep, and smoking, age-related differences, rank, duty type, shift work characteristics, socioeconomic variables and metabolic markers were not included. Information on menstrual cycle phase was not collected, which may have influenced body composition measurements among female participants. Finally, body composition was assessed using bioelectrical impedance analysis, which, although suitable for this type of study, is less precise than reference methods.

## 6. Conclusions

The study identified several suboptimal dietary behaviours and a high prevalence of excess body weight and adiposity among Polish police officers. As one of the occupational groups examined within the National Health Programme, police officers demonstrated suboptimal food consumption frequency patterns similar to those previously observed in other Polish uniformed services, particularly with regard to the low consumption of several health-promoting food groups and the high prevalence of excess adiposity. The findings may support the development of occupational health and nutritional education strategies tailored to police personnel. The discrepancy between BMI- and FMI-based classification additionally highlights the importance of incorporating body composition assessment into routine evaluation of nutritional status in physically active occupational groups. Further studies including broader lifestyle-related variables are warranted to better understand the determinants of nutritional status in this occupational group.

## Figures and Tables

**Table 1:** Frequency of food consumption among Polish police officers.

Food Groups	Percentage of Consumption Frequency							
	1	2	3	4	5	6	7	8
Fruits, vegetables and potatoes								
Fruits together—all types	1	1	3	10	8	49	23	6
Stone fruits	1	6	14	32	22	22	3	0
Kiwi fruit and citrus	1	4	12	34	19	24	5	1
Other tropical fruits	5	14	28	30	13	9	1	0
Bananas	1	2	5	18	23	37	12	2
Apples and pears	2	2	5	19	19	41	9	2
Avocado	30	20	22	14	7	7	1	0
Olives	25	21	19	17	8	8	2	0
Dried fruits	14	21	21	20	12	10	2	0
Sweet fruit preserves and candied fruits	10	19	20	27	10	12	2	0
Vegetables—all types	0	1	2	10	10	43	27	7
Crucifers	0	6	9	32	26	23	3	1
Yellow–orange vegetables	0	1	5	21	26	39	7	0
Green leafy vegetables	2	4	8	29	22	26	7	1
Tomatoes	1	0	2	14	18	46	18	2
Vegetables: fresh cucumbers, squash, zucchini, pumpkin	0	3	3	22	20	42	8	1
Root vegetables and others	0	3	6	28	21	32	9	0
Potatoes in various forms	1	2	5	15	18	43	15	1
Seeds of legumes								
Fresh seeds of legumes and canned ones	2	15	19	38	16	10	1	0
Dry seeds of legumes	7	20	29	25	13	7	0	0
Cereal products								
Wholemeal or with grains, so-called dark bread	2	3	6	15	8	34	28	3
Refined bread, so-called white bread	2	3	7	15	14	30	25	4
Unrefined groats coarse	3	6	10	28	18	29	6	0
Refined cereal grain	3	5	11	24	22	30	4	1
Ready-to-eat breakfast cereal products	12	12	17	21	13	22	3	0
Dairy products and eggs								
Milk and milk drinks	2	2	6	15	12	37	21	5
Sweetened milk drinks	7	6	16	16	19	26	9	1
Cottage cheese	2	2	8	18	19	40	11	0
Flavoured cottage cheese	18	13	17	19	18	12	3	0
Cheese	1	2	5	22	16	43	10	2
Eggs and egg dishes	1	0	4	14	22	49	9	2
Meat products								
Sausages, different types	4	1	8	15	19	38	13	3
High-quality cold cuts	2	1	2	11	15	49	16	2
Sausage products and offal	11	14	19	22	15	17	1	0
Red meat	4	5	10	21	24	32	4	0
Poultry and rabbit	3	4	4	17	21	43	6	2
Wild game meat	34	33	12	12	4	4	0	0
Fish								
Lean fish	7	12	25	29	20	7	1	0
Oily fish	7	17	23	29	16	7	0	0
Fats								
Oil, all kinds	1	3	9	21	15	37	13	2
Butter, all types	3	3	5	9	7	30	37	6
Margarine, all types	31	10	15	14	5	15	8	1
Cream, sweet or sour cream, for food or beverages	10	12	13	24	15	22	4	0
Other animal fats	30	18	17	20	8	6	1	0
Mayonnaise and dressings, i.e., salad dressings—all types	7	11	14	33	15	17	2	0
Nuts and grains								
Nuts	2	10	17	28	21	18	4	0
Grains	8	15	22	22	15	14	3	1
Sweets and snacks								
Sugar to sweeten beverages	23	7	6	11	7	15	26	6
Honey to sweeten food and beverages	21	19	19	16	5	13	5	2
Chocolate, chocolate candies, and candy bars	1	5	10	23	26	26	6	2
Non-chocolate candies	14	16	23	23	11	11	2	1
Biscuits and cakes	5	8	18	30	19	16	2	1
Ice cream and pudding	4	18	29	30	14	5	0	0
Salty snacks	8	11	21	32	15	11	1	0
Soft drinks								
Fruit juices and fruit nectars	5	8	13	21	13	32	7	1
Vegetable juices and vegetable-fruit ones	9	17	21	23	15	13	2	0
Energy drinks	41	13	13	16	7	8	2	0
Sweetened sodas such as Fanta, Coca-Cola, Mirinda, Sprite	17	9	20	20	15	16	2	1
Alcoholic beverages								
Beer	10	9	14	26	18	22	1	0
Wine and drinks	17	14	25	24	14	5	0	0
Vodka and spirits	14	17	26	30	10	2	1	0

Column numbers correspond to the following consumption frequency categories: 1—never or almost never; 2—once a quarter or less often; 3—once a month or less often; 4—a few times a month; 5—once a week; 6—several times a week; 7—every day; 8—several times a day.

**Table 2:** Comparison of food consumption frequency between male and female police officers.

Groups of Products	Male Police Officers		Female Police Officers			
	X ± SD	Me	X ± SD	Me	*p*	r
Food groups						
Fruits, vegetables and potatoes	5.33 ± 3.17	5.11	7.44 ± 3.43	7.58	<0.001 *	0.24
Seeds of legumes	0.21 ± 0.28	0.11	0.24 ± 0.33	0.16	0.490	0.04
Cereal products	1.86 ± 0.97	1.81	1.84 ± 0.89	1.88	0.943	0.01
Dairy products and eggs	2.24 ± 1.38	2.13	2.22 ± 1.52	2.08	0.678	0.03
Meat products	1.84 ± 1.10	1.80	1.66 ± 1.18	1.37	0.122	0.09
Fish	0.21 ± 0.28	0.13	0.18 ± 0.26	0.11	0.151	0.09
Fats	1.77 ± 1.33	1.53	1.79 ± 1.09	1.55	0.706	0.03
Nuts and grains	0.36 ± 0.45	0.16	0.47 ± 0.52	0.17	0.184	0.08
Sweets and snacks	1.50 ± 1.20	1.33	1.69 ± 2.25	1.18	0.671	0.02
Non-alcoholic beverages	0.76 ± 0.71	0.61	0.57 ± 0.75	0.23	0.001 *	0.20
Alcoholic beverages	0.37 ± 0.36	0.24	0.14 ± 0.18	0.09	<0.001 *	0.33
Selected products						
Fruits, vegetables and potatoes						
Fruits together—all types	0.59 ± 0.41	0.57	0.92 ± 0.57	1.00	<0.001 *	0.26
Stone fruits	0.21 ± 0.27	0.08	0.30 ± 0.29	0.14	0.018 *	0.13
Kiwi fruit and citrus	0.25 ± 0.32	0.08	0.32 ± 0.31	0.14	0.154	0.07
Other tropical fruits	0.11 ± 0.16	0.08	0.14 ± 0.23	0.08	0.209	0.07
Bananas	0.41 ± 0.36	0.57	0.37 ± 0.44	0.14	0.459	0.05
Apples and pears	0.38 ± 0.36	0.14	0.56 ± 0.49	0.57	0.018 *	0.14
Avocado	0.07 ± 0.14	0.01	0.10 ± 0.23	0.03	0.899	0.02
Olives	0.09 ± 0.17	0.03	0.14 ± 0.28	0.03	0.879	0.03
Dried fruits	0.12 ± 0.24	0.03	0.16 ± 0.25	0.08	0.076	0.12
Sweet fruit preserves and candied fruits	0.14 ± 0.25	0.08	0.11 ± 0.21	0.03	0.066	0.11
Vegetables—all types	0.62 ± 0.45	0.57	0.97 ± 0.54	1.00	<0.001 *	0.28
Crucifers	0.23 ± 0.29	0.14	0.30 ± 0.31	0.14	0.210	0.09
Yellow–orange vegetables	0.33 ± 0.29	0.14	0.48 ± 0.33	0.57	0.004 *	0.18
Green leafy vegetables	0.25 ± 0.31	0.14	0.53 ± 0.42	0.57	<0.001 *	0.28
Tomatoes	0.46 ± 0.34	0.57	0.71 ± 0.42	0.57	<0.001 *	0.25
Vegetables: fresh cucumbers, squash, zucchini, pumpkin	0.36 ± 0.34	0.14	0.55 ± 0.33	0.57	<0.001 *	0.21
Root vegetables and others	0.31 ± 0.31	0.14	0.47 ± 0.37	0.57	0.002 *	0.18
Potatoes in various forms	0.45 ± 0.35	0.57	0.46 ± 0.36	0.57	0.941	0.01
Seeds of legumes						
Fresh seeds of legumes and canned ones	0.12 ± 0.17	0.08	0.15 ± 0.22	0.08	0.692	0.03
Dry seeds of legumes	0.09 ± 0.14	0.03	0.09 ± 0.13	0.08	0.496	0.04
Cereal products						
Wholemeal or with grains, so-called dark bread	0.55 ± 0.45	0.57	0.65 ± 0.47	0.57	0.156	0.07
Refined bread, so-called white bread	0.55 ± 0.46	0.57	0.42 ± 0.53	0.14	0.010 *	0.16
Unrefined groats coarse	0.27 ± 0.28	0.14	0.30 ± 0.33	0.14	0.368	0.05
Refined cereal grain	0.29 ± 0.32	0.14	0.24 ± 0.27	0.14	0.448	0.04
Ready-to-eat breakfast cereal products	0.20 ± 0.26	0.08	0.22 ± 0.30	0.08	0.881	0.01
Dairy products and eggs						
Milk and milk drinks	0.54 ± 0.48	0.57	0.57 ± 0.41	0.57	0.393	0.05
Sweetened milk drinks	0.31 ± 0.33	0.14	0.26 ± 0.42	0.03	0.007 *	0.17
Cottage cheese	0.38 ± 0.32	0.57	0.39 ± 0.30	0.57	0.670	0.03
Flavoured cottage cheese	0.16 ± 0.24	0.08	0.09 ± 0.18	0.03	0.019 *	0.16
Cheese	0.41 ± 0.35	0.57	0.46 ± 0.46	0.57	0.926	0.01
Eggs and egg dishes	0.44 ± 0.32	0.57	0.45 ± 0.45	0.36	0.716	0.03
Meat products						
Sausages, different types	0.44 ± 0.40	0.57	0.46 ± 0.48	0.57	0.390	0.05
High-quality cold cuts	0.53 ± 0.38	0.57	0.47 ± 0.40	0.57	0.226	0.07
Sausage products and offal	0.17 ± 0.23	0.08	0.08 ± 0.16	0.03	<0.001 *	0.25
Red meat	0.28 ± 0.27	0.14	0.25 ± 0.27	0.14	0.341	0.06
Poultry and rabbit	0.39 ± 0.34	0.57	0.37 ± 0.39	0.14	0.474	0.04
Wild game meat	0.06 ± 0.13	0.01	0.03 ± 0.12	0.00	<0.001 *	0.23
Fish						
Lean fish	0.11 ± 0.16	0.08	0.11 ± 0.17	0.03	0.089	0.10
Oily fish	0.11 ± 0.16	0.08	0.08 ± 0.11	0.03	0.163	0.09
Fats						
Oil, all kinds	0.40 ± 0.39	0.14	0.53 ± 0.46	0.57	0.045 *	0.13
Butter, all types	0.66 ± 0.49	0.57	0.71 ± 0.56	0.57	0.771	0.02
Margarine, all types	0.23 ± 0.36	0.03	0.08 ± 0.20	0.01	0.003 *	0.17
Cream, sweet or sour cream, for food or beverages	0.21 ± 0.28	0.08	0.26 ± 0.31	0.08	0.876	0.02
Other animal fats	0.09 ± 0.17	0.03	0.07 ± 0.30	0.00	<0.001 *	0.24
Mayonnaise and dressings, i.e., salad dressings—all types	0.19 ± 0.27	0.08	0.13 ± 0.21	0.08	0.054	0.12
Nuts and grains						
Nuts	0.21 ± 0.28	0.08	0.20 ± 0.26	0.08	0.594	0.04
Grains	0.16 ± 0.26	0.08	0.27 ± 0.38	0.08	0.004 *	0.19
Sweets and snack						
Sugar to sweeten beverages	0.52 ± 0.57	0.57	0.30 ± 0.51	0.03	0.002 *	0.18
Honey to sweeten food and beverages	0.18 ± 0.34	0.03	0.23 ± 0.41	0.03	0.968	0.00
Chocolate, chocolate candies, and candy bars	0.29 ± 0.31	0.14	0.44 ± 0.61	0.14	0.610	0.05
Non-chocolate candies	0.13 ± 0.21	0.03	0.17 ± 0.43	0.06	0.924	0.01
Biscuits and cakes	0.17 ± 0.23	0.08	0.28 ± 0.44	0.08	0.109	0.11
Ice cream and pudding	0.08 ± 0.11	0.03	0.14 ± 0.32	0.03	0.705	0.01
Salty snacks	0.14 ± 0.20	0.08	0.13 ± 0.31	0.08	0.132	0.09
Soft drinks						
Fruit juices and fruit nectars	0.34 ± 0.36	0.14	0.19 ± 0.28	0.03	<0.001 *	0.22
Vegetable juices and vegetable-fruit ones	0.14 ± 0.20	0.08	0.18 ± 0.37	0.03	0.191	0.08
Energy drinks	0.09 ± 0.18	0.01	0.11 ± 0.28	0.00	0.003 *	0.19
Sweetened sodas such as Fanta, Coca-Cola, Mirinda, Sprite	0.19 ± 0.27	0.08	0.09 ± 0.31	0.01	<0.001 *	0.30
Alcoholic beverages						
Beer	0.21 ± 0.23	0.08	0.06 ± 0.12	0.03	<0.001 *	0.35
Wine and drinks	0.08 ± 0.12	0.03	0.06 ± 0.12	0.03	0.004 *	0.18
Vodka and spirits	0.08 ± 0.14	0.03	0.02 ± 0.03	0.01	<0.001 *	0.30

* Mann–Whitney U Test * *p* < 0.05; X—mean; SD—standard deviation; Me—median, r—effect size. Food consumption frequency categories were converted into daily frequency values as follows (range: 0.003–2): never or almost never—0.003, once a quarter or less often—0.01, once a month or less often—0.03, a few times a month—0.08, once a week—0.14, several times a week—0.57, every day—1, several times a day—2. *p*-values remaining statistically significant after FDR correction are shown in bold.

**Table 3:** Nutritional status indicators among Polish police officers.

Percentage of Classifications	
BMI:	
Underweight	0
Normal body weight	33
Excessive body weight	67
FMI:	
Fat deficit	2
Normal fat	16
Excess fat	82
V FAT L:	
Healthy level	95
Excess level	5

BMI—body mass index, FMI—fat mass index, V FAT L—visceral fat level.

## Data Availability

The data presented in this study are available upon request from the corresponding author. The data are not publicly available due to privacy and ethical restrictions.

## Supplementary Materials

The following supporting information can be downloaded at: https://www.mdpi.com/article/10.3390/nu18142385/s1, Table S1: Correlations between food consumption frequency and BMI, FMI, FAT%, and V FAT L among male and female police officers. Table S2. Adjusted *p*-values obtained using the Benjamini–Hochberg false discovery rate procedure.

## Abbreviations

The following abbreviations are used in this manuscript:

BMI	Body Mass Index
FAT%	Fat body percentage
FFQ	Food frequency questionnaire
FMI	Fat Mass Index
V FAT L	Visceral fat level
