# Supplementary material for: Dietary Habits and Nutritional Status Among Polish Police Officers: A Cross-Sectional Study Within the National Health Programme
Source: Nutrients. 2026 Jul 22;18(14):2385. doi: 10.3390/nu18142385 (PMC13415224; doi:10.3390/nu18142385)
Supplement: Supplementary file 1 [file nutrients-18-02385-s001.zip › nutrients-4411276-supplementary.pdf]

Table S1. Correlations between food consumption frequency and BMI, FMI, FAT%, and V FAT L among male and female police officers.

Groups of products	Male police officers				Female police officers			
	BMI	FMI	FAT%	V FAT L	BMI	FMI	FAT%	V FAT L
	Rho<br>*p*	Rho<br>*p*	Rho<br>*p*	Rho<br>*p*	Rho<br>*p*	Rho<br>*p*	Rho<br>*p*	Rho<br>*p*
**Food groups**								
Fruits, vegetables and potatoes	-0.036<br>0.595	-0.067<br>0.326	-0.087<br>0.204	-0.036<br>0.598	0.058<br>0.702	0.040<br>0.792	0.065<br>0.668	0.185<br>0.217
Seeds of legumes	0.101<br>0.139	0.064<br>0.351	0.053<br>0.438	0.088<br>0.199	-0.190<br>0.205	-0.247<br>0.098	-0.232<br>0.121	-0.136<br>0.368
Cereal products	-0.054<br>0.428	-0.098<br>0.152	-0.108<br>0.114	-0.139*<br>0.042	-0.199<br>0.185	-0.302*<br>0.041	-0.324*<br>0.028	-0.278<br>0.062
Dairy products and eggs	0.002<br>0.974	-0.045<br>0.509	-0.083<br>0.223	-0.061<br>0.375	-0.095<br>0.529	-0.045<br>0.767	0.008<br>0.957	0.032<br>0.833
Meat products	0.014<br>0.835	0.031<br>0.647	0.025<br>0.720	-0.019<br>0.776	-0.023<br>0.878	0.010<br>0.947	0.020<br>0.896	0.037<br>0.807
Fish	0.073<br>0.284	0.027<br>0.689	0.004<br>0.955	0.014<br>0.842	-0.193<br>0.199	-0.205<br>0.172	-0.190<br>0.207	-0.167<br>0.268
Fats	-0.142*<br>0.037	-0.090<br>0.188	-0.077<br>0.258	-0.121<br>0.075	-0.011<br>0.943	0.016<br>0.914	0.078<br>0.605	0.093<br>0.537
Nuts and grains	0.125<br>0.067	0.061<br>0.376	0.026<br>0.701	0.083<br>0.224	-0.351*<br>0.017	-0.287<br>0.053	-0.259<br>0.083	-0.232<br>0.121
Sweets and snacks	-0.140*<br>0.039	-0.064<br>0.351	-0.026<br>0.700	-0.064<br>0.351	-0.146<br>0.334	-0.105<br>0.488	-0.062<br>0.682	-0.017<br>0.910
Non-alcoholic beverages	-0.049<br>0.475	-0.027<br>0.689	-0.021<br>0.760	-0.091<br>0.183	0.129<br>0.394	0.152<br>0.314	0.119<br>0.432	0.093<br>0.537
Alcoholic beverages	0.077<br>0.262	0.125<br>0.066	0.139*<br>0.042	0.093<br>0.173	-0.139<br>0.358	-0.169<br>0.262	-0.192<br>0.202	-0.209<br>0.163
**Selected products**								
Fruits, vegetables, seeds of legumes, and potatoes								
Fruits together-all types	-0.098<br>0.157	-0.125<br>0.071	-0.153*<br>0.027	-0.018<br>0.792	-0.044<br>0.775	-0.010<br>0.949	0.033<br>0.833	0.157<br>0.310
Stone fruits	-0.018<br>0.787	-0.074<br>0.279	-0.107<br>0.118	-0.051<br>0.452	0.082<br>0.588	0.122<br>0.420	0.103<br>0.495	0.203<br>0.177
Kiwi fruit and citrus	-0.083<br>0.228	-0.100<br>0.145	-0.114<br>0.096	-0.113<br>0.100	0.029<br>0.852	0.027<br>0.862	0.003<br>0.982	0.089<br>0.560
Other tropical fruits	-0.020<br>0.773	-0.045<br>0.510	-0.052<br>0.445	-0.060<br>0.379	-0.065<br>0.671	-0.020<br>0.897	0.005<br>0.974	0.103<br>0.499
Bananas	-0.112<br>0.100	-0.130<br>0.056	-0.130<br>0.057	-0.065<br>0.341	0.121<br>0.422	0.081<br>0.593	0.036<br>0.812	0.036<br>0.812
Apples and pears	0.030<br>0.666	0.012<br>0.861	0.007<br>0.924	0.070<br>0.305	-0.023<br>0.882	-0.029<br>0.848	-0.004<br>0.977	0.024<br>0.873
Avocado	0.133	0.102	0.070	0.073	-0.160	-0.185	-0.217	-0.135

	0.051	0.137	0.307	0.289	0.300	0.228	0.156	0.381
Olives	0.200*	0.146*	0.103	0.184*	0.034	0.008	-0.006	0.063
	0.003	0.032	0.133	0.007	0.825	0.958	0.970	0.679
Dried fruits	0.070	0.015	-0.011	0.064	-0.048	-0.073	-0.032	0.036
	0.308	0.826	0.870	0.352	0.753	0.631	0.832	0.810
Sweet fruit preserves and candied fruits	-0.187*	-0.162*	-0.136*	-0.150*	0.064	-0.045	-0.103	-0.110
	0.006	0.017	0.046	0.028	0.672	0.765	0.496	0.468
Vegetables-all types	-0.094	-0.106	-0.113	-0.113	-0.366*	-0.434*	-0.373*	-0.226
	0.174	0.128	0.103	0.104	0.016	0.004	0.014	0.145
Crucifers	0.026	-0.036	-0.078	-0.062	0.083	0.070	0.101	0.195
	0.705	0.604	0.256	0.367	0.582	0.646	0.505	0.194
Yellow–orange vegetables	-0.076	-0.097	-0.104	-0.119	-0.060	-0.095	-0.058	0.047
	0.265	0.157	0.128	0.080	0.694	0.531	0.703	0.754
Green leafy vegetables	0.076	0.029	-0.001	0.044	0.076	0.071	0.111	0.223
	0.269	0.678	0.988	0.519	0.618	0.643	0.467	0.141
Tomatoes	0.000	-0.033	-0.051	0.010	0.187	0.113	0.092	0.201
	0.997	0.639	0.468	0.885	0.212	0.455	0.543	0.181
Vegetables: fresh cucumbers, squash, zucchini, pumpkin	0.022	-0.023	-0.044	-0.086	0.055	-0.028	-0.048	0.096
	0.753	0.736	0.521	0.208	0.719	0.855	0.755	0.531
Root vegetables and others	0.043	0.011	-0.014	0.074	0.036	-0.021	0.010	0.129
	0.529	0.878	0.836	0.279	0.810	0.888	0.947	0.392
Potatoes in various forms	-0.112	-0.073	-0.042	-0.091	0.116	0.144	0.210	0.166
	0.100	0.286	0.536	0.185	0.445	0.340	0.161	0.271
Seeds of legumes								
Fresh seeds of legumes and canned ones	0.070	0.020	0.009	0.034	-0.172	-0.238	-0.237	-0.156
	0.305	0.766	0.899	0.623	0.253	0.111	0.112	0.300
Dry seeds of legumes	0.118	0.094	0.085	0.134*	-0.130	-0.164	-0.145	-0.048
	0.085	0.168	0.212	0.050	0.388	0.276	0.337	0.754
Cereal products								
Wholemeal or with grains, so-called dark bread	-0.070	-0.110	-0.130	-0.085	-0.225	-0.353*	-0.421*	-0.363*
	0.306	0.107	0.057	0.211	0.133	0.016	0.004	0.013
Refined bread, so-called white bread	-0.089	-0.046	-0.028	-0.057	-0.005	0.017	0.052	0.010
	0.191	0.500	0.684	0.406	0.975	0.911	0.732	0.947
Unrefined groats coarse	-0.009	-0.084	-0.117	-0.144*	-0.218	-0.254	-0.253	-0.180
	0.893	0.221	0.087	0.034	0.146	0.089	0.089	0.232
Refined cereal grain	-0.021	-0.092	-0.107	-0.143*	0.018	-0.016	0.020	0.001
	0.756	0.179	0.118	0.035	0.905	0.914	0.894	0.994
Ready-to-eat breakfast cereal products	0.054	0.029	0.008	-0.054	-0.076	-0.079	-0.063	-0.147
	0.431	0.672	0.911	0.430	0.614	0.601	0.677	0.331
Dairy products and eggs								
Milk and milk drinks	0.037	-0.020	-0.069	-0.005	-0.115	0.002	0.082	0.081
	0.594	0.767	0.312	0.940	0.446	0.988	0.589	0.593
Sweetened milk drinks	-0.065	-0.071	-0.082	-0.116	0.045	-0.066	-0.107	-0.128
	0.340	0.299	0.230	0.089	0.768	0.663	0.479	0.397

Cottage cheese	0.005<br>0.940	-0.074<br>0.280	-0.113<br>0.098	-0.036<br>0.603	0.109<br>0.469	0.119<br>0.430	0.145<br>0.335	0.175<br>0.244
Flavored cottage cheese	-0.003<br>0.966	-0.019<br>0.782	-0.036<br>0.598	-0.067<br>0.327	0.060<br>0.694	-0.073<br>0.630	-0.136<br>0.367	-0.202<br>0.179
Cheese	-0.069<br>0.314	-0.070<br>0.309	-0.076<br>0.264	-0.098<br>0.152	0.117<br>0.439	0.119<br>0.432	0.138<br>0.361	0.137<br>0.363
Eggs and egg dishes	0.052<br>0.446	0.012<br>0.863	-0.008<br>0.908	-0.016<br>0.812	-0.131<br>0.387	-0.100<br>0.510	-0.067<br>0.660	-0.073<br>0.630
Meat products								
Sausages, different types	-0.002<br>0.978	0.027<br>0.690	0.023<br>0.739	0.028<br>0.682	0.018<br>0.908	0.002<br>0.992	-0.009<br>0.951	0.015<br>0.923
High-quality cold cuts	-0.017<br>0.799	0.008<br>0.910	0.009<br>0.894	0.013<br>0.847	-0.155<br>0.304	-0.111<br>0.463	-0.059<br>0.698	-0.004<br>0.980
Sausage products and offal	0.022<br>0.747	0.066<br>0.332	0.076<br>0.269	0.032<br>0.636	-0.010<br>0.946	0.033<br>0.828	0.068<br>0.656	0.024<br>0.875
Red meat	0.044<br>0.522	0.054<br>0.429	0.051<br>0.455	-0.002<br>0.978	0.177<br>0.239	0.241<br>0.106	0.251<br>0.092	0.266<br>0.074
Poultry and rabbit	0.059<br>0.390	0.016<br>0.823	-0.009<br>0.895	-0.014<br>0.838	0.045<br>0.767	0.086<br>0.571	0.083<br>0.585	0.009<br>0.953
Wild game meat	0.139*<br>0.042	0.120<br>0.081	0.105<br>0.126	0.017<br>0.801	0.234<br>0.121	0.214<br>0.158	0.165<br>0.279	0.126<br>0.410
Fish								
Lean fish	0.061<br>0.376	0.023<br>0.736	0.007<br>0.919	0.011<br>0.873	-0.160<br>0.288	-0.183<br>0.224	-0.175<br>0.244	-0.145<br>0.335
Oily fish	0.082<br>0.231	0.032<br>0.636	0.003<br>0.968	0.015<br>0.823	-0.299*<br>0.044	-0.280<br>0.059	-0.238<br>0.111	-0.233<br>0.120
Fats								
Oil, all kinds	-0.003<br>0.968	0.021<br>0.760	0.019<br>0.779	-0.006<br>0.931	0.028<br>0.854	0.084<br>0.581	0.153<br>0.309	0.225<br>0.133
Butter, all types	-0.094<br>0.169	-0.042<br>0.541	-0.020<br>0.770	-0.039<br>0.575	-0.203<br>0.176	-0.199<br>0.186	-0.134<br>0.373	-0.161<br>0.286
Margarine, all types	-0.073<br>0.285	-0.046<br>0.503	-0.044<br>0.524	-0.114<br>0.095	-0.158<br>0.295	-0.262<br>0.079	-0.323*<br>0.029	-0.341*<br>0.021
Cream, sweet or sour cream, for food or beverages	-0.119<br>0.081	-0.079<br>0.248	-0.079<br>0.250	-0.063<br>0.356	0.232<br>0.121	0.234<br>0.118	0.296*<br>0.046	0.315*<br>0.033
Other animal fats	-0.048<br>0.480	-0.010<br>0.887	-0.010<br>0.885	-0.041<br>0.546	-0.018<br>0.904	-0.056<br>0.710	-0.065<br>0.667	-0.127<br>0.399
Mayonnaise and dressings, i. .e., salad dressings-all types	0.006<br>0.930	0.035<br>0.613	0.015<br>0.822	-0.043<br>0.530	-0.024<br>0.872	-0.013<br>0.932	0.019<br>0.900	-0.035<br>0.818
Nuts and grains								
Nuts	0.119<br>0.083	0.056<br>0.413	0.026<br>0.701	0.068<br>0.321	-0.346*<br>0.018	-0.332*<br>0.024	-0.273<br>0.067	-0.180<br>0.232

Grains	0.132<br>0.053	0.074<br>0.280	0.041<br>0.555	0.077<br>0.264	-0.276<br>0.066	-0.201<br>0.185	-0.191<br>0.209	-0.164<br>0.280
Sweets and snacks								
Sugar to sweeten beverages	-0.157*<br>0.021	-0.065<br>0.339	-0.032<br>0.641	-0.063<br>0.354	0.016<br>0.916	0.037<br>0.805	0.063<br>0.679	0.023<br>0.880
Honey to sweeten food and beverages	-0.013<br>0.844	-0.030<br>0.659	-0.031<br>0.646	0.039<br>0.569	-0.069<br>0.650	-0.068<br>0.651	-0.043<br>0.776	-0.005<br>0.974
Chocolate, chocolate candies, and candy bars	-0.139*<br>0.041	-0.096<br>0.162	-0.060<br>0.384	-0.125<br>0.066	-0.086<br>0.569	-0.115<br>0.448	-0.093<br>0.541	-0.056<br>0.710
Non-chocolate candies	-0.122<br>0.074	-0.102<br>0.137	-0.095<br>0.163	-0.119<br>0.082	0.262<br>0.078	0.163<br>0.279	0.116<br>0.443	0.084<br>0.578
Biscuits and cakes	-0.163*<br>0.016	-0.141*<br>0.038	-0.125<br>0.067	-0.138*<br>0.043	0.030<br>0.845	-0.001<br>0.994	0.006<br>0.971	0.022<br>0.883
Ice cream and pudding	0.015<br>0.827	-0.033<br>0.626	-0.057<br>0.403	-0.098<br>0.152	0.063<br>0.677	-0.053<br>0.724	-0.118<br>0.435	-0.153<br>0.309
Salty snacks	0.054<br>0.428	0.066<br>0.335	0.065<br>0.338	-0.093<br>0.173	-0.106<br>0.482	-0.171<br>0.256	-0.215<br>0.151	-0.266<br>0.074
Soft drinks								
Fruit juices and fruit nectars	-0.142*<br>0.037	-0.092<br>0.178	-0.077<br>0.262	-0.127<br>0.061	0.039<br>0.800	0.046<br>0.766	-0.001<br>0.995	-0.016<br>0.918
Vegetable juices and vegetable-fruit ones	0.070<br>0.306	0.052<br>0.450	0.046<br>0.500	0.035<br>0.604	-0.021<br>0.892	0.030<br>0.841	0.049<br>0.747	0.063<br>0.676
Energy drinks	0.109<br>0.111	0.049<br>0.471	0.015<br>0.823	-0.057<br>0.405	-0.033<br>0.831	-0.137<br>0.369	-0.189<br>0.213	-0.237<br>0.117
Sweetened sodas such as Fanta, Coca-Cola, Mirinda, Sprite	0.085<br>0.215	0.102<br>0.138	0.110<br>0.108	0.011<br>0.875	0.148<br>0.333	0.038<br>0.806	-0.023<br>0.879	-0.025<br>0.870
Alcoholic beverages								
Beer	0.023<br>0.740	0.069<br>0.316	0.083<br>0.224	0.054<br>0.431	-0.272<br>0.071	-0.337*<br>0.023	-0.370*<br>0.012	-0.375*<br>0.011
Wine and drinks	0.091<br>0.184	0.096<br>0.160	0.088<br>0.199	0.024<br>0.726	-0.085<br>0.576	-0.089<br>0.558	-0.117<br>0.440	-0.122<br>0.420
Vodka and spirits	0.181*<br>0.008	0.231*<br>0.001	0.230*<br>**0.001**	0.158*<br>0.020	-0.189<br>0.208	-0.229<br>0.126	-0.236<br>0.114	-.298*<br>0.044

BMI - body mass index, FMI - fat mass index, FAT% - body fat percentage, V FAT L - visceral fat level
*Rho for $p < 0.05$. p-values remaining statistically significant after FDR correction are shown in bold

Table S2. Adjusted *p*-values obtained using the Benjamini–Hochberg false discovery rate procedure.

Groups of products	*p*-adj1	*p*-adj2	*p*-adj3	*p*-adj4	*p*-adj5	*p*-adj6	*p*-adj7	*p*-adj8	*p*-adj9
Food groups									
Fruits, vegetables and potatoes	<0.001*	0.727	0.897	0.748	0.731	0.858	0.968	0.919	0.597
Seeds of legumes	0.770	0.382	0.772	0.803	0.438	0.564	0.359	0.444	0.675
Cereal products	0.943	0.673	0.836	0.627	0.462	1.000	0.451	0.308	0.682
Dairy products and eggs	0.829	0.974	0.700	0.613	0.516	0.727	1.000	0.957	0.916
Meat products	0.336	0.919	0.791	0.880	0.854	0.966	0.947	0.986	0.986
Fish	0.332	0.521	0.758	0.955	0.842	0.730	0.473	0.455	0.590
Fats	0.777	0.407	0.689	0.568	0.413	0.943	1.000	0.951	0.844
Nuts and grains	0.337	0.246	0.591	0.964	0.411	0.187	0.292	0.457	0.666
Sweets and snacks	0.923	0.215	0.644	1.000	0.552	0.735	0.767	0.834	0.910
Non-alcoholic beverages	0.004*	0.653	0.689	0.836	0.503	0.619	0.576	0.792	0.738
Alcoholic beverages	<0.001*	0.576	0.726	0.462	0.634	0.656	0.576	0.556	0.598
Selected products									
Fruits, vegetables and potatoes									
Fruits together-all types	<0.001*	0.532	0.722	1.000	0.966	1.000	1.000	1.000	0.756
Stone fruits	0.050	0.941	0.774	0.654	0.726	1.000	1.000	1.000	0.831
Kiwi fruit and citrus	0.294	0.580	0.680	0.837	0.436	1.000	1.000	0.998	0.899
Other tropical fruits	0.354	0.943	0.819	0.876	0.701	1.000	1.000	1.000	0.846
Bananas	0.636	0.407	0.683	1.000	0.743	1.000	1.000	1.000	1.000
Apples and pears	0.048*	0.923	0.921	0.972	0.744	0.996	1.000	1.000	1.000
Avocado	0.962	0.346	0.836	0.720	0.735	1.000	0.869	0.732	0.801
Olives	0.975	0.183	0.651	0.541	0.427	1.000	1.000	1.000	0.963
Dried fruits	0.166	0.587	0.900	1.000	0.740	1.000	1.000	1.000	1.000
Sweet fruit preserves and candied fruits	0.149	0.183	0.519	1.000	0.569	1.000	1.000	0.976	0.816
Vegetables-all types	<0.001*	0.531	0.868	0.698	0.423	0.976	0.244	0.285	0.737
Crucifers	0.346	0.956	0.921	0.710	0.700	1.000	1.000	0.963	0.740
Yellow–orange vegetables	0.014*	0.622	0.684	0.558	0.488	1.000	1.000	0.997	1.000
Green leafy vegetables	<0.001*	0.608	0.880	1.000	0.812	1.000	1.000	1.000	0.782

Tomatoes	<0.001*	0.997	0.886	0.865	0.931	0.995	1.000	0.974	0.736
Vegetables: fresh cucumbers, squash, zucchini, pumpkin	<0.001*	0.957	0.916	0.908	0.634	1.000	1.000	1.000	0.875
Root vegetables and others	0.009*	0.768	0.908	1.000	0.740	1.000	1.000	1.000	0.797
Potatoes in various forms	0.957	0.381	0.698	0.884	0.594	1.000	1.000	0.702	0.827
Seeds of legumes									
Fresh seeds of legumes and canned ones	0.844	0.642	0.899	1.000	0.826	1.000	0.752	0.683	0.796
Dry seeds of legumes	0.658	0.370	0.603	0.718	0.436	1.000	0.935	0.979	1.000
Cereal products									
Wholemeal or with grains, so-called dark bread	0.288	0.622	0.816	0.869	0.613	0.901	0.488	0.244	0.397
Refined bread, so-called white bread	0.029*	0.530	0.847	0.993	0.708	0.975	1.000	1.000	1.000
Unrefined groats coarse	0.561	0.990	0.674	0.885	0.519	0.891	0.776	0.776	0.832
Refined cereal grain	0.636	0.941	0.575	0.600	0.427	0.969	1.000	1.000	0.994
Ready-to-eat breakfast cereal products	0.960	0.692	0.891	0.992	0.729	1.000	1.000	1.000	0.777
Dairy products and eggs									
Milk and milk drinks	0.571	0.843	0.883	0.705	0.956	1.000	1.000	0.998	0.904
Sweetened milk drinks	0.021*	0.610	0.702	0.702	0.452	1.000	1.000	1.000	0.781
Cottage cheese	0.834	1.000	0.743	0.747	0.836	1.000	1.000	1.000	0.783
Flavored cottage cheese	0.048*	1.000	0.883	0.935	0.739	1.000	1.000	0.973	0.780
Cheese	0.957	0.580	0.698	0.671	0.580	1.000	1.000	1.000	0.791
Eggs and egg dishes	0.840	0.698	0.908	1.000	0.953	1.000	1.000	1.000	0.937
Meat products									
Sausages, different types	0.580	0.994	0.877	1.000	0.867	0.955	1.000	1.000	1.000
High-quality cold cuts	0.363	0.937	0.910	1.000	0.939	0.976	1.000	1.000	0.996
Sausage products and offal	<0.001*	0.970	0.698	0.656	0.825	0.962	1.000	1.000	1.000
Red meat	0.533	0.777	0.793	0.867	0.978	1.000	0.808	0.702	0.752
Poultry and rabbit	0.643	0.661	0.913	1.000	0.947	1.000	1.000	1.000	1.000
Wild game meat	<0.001*	0.320	0.706	0.591	0.958	1.000	0.803	0.946	0.758
Fish									

Lean fish	0.187	0.655	0.898	0.983	0.951	1.000	0.911	0.876	0.757
Oily fish	0.292	0.564	0.902	1.000	0.947	0.895	0.720	0.752	0.813
Fats									
Oil, all kinds	0.110	1.000	0.909	1.000	0.963	1.000	1.000	0.992	0.811
Butter, all types	0.887	0.543	0.846	1.000	0.816	0.976	0.810	0.948	0.793
Margarine, all types	0.012*	0.621	0.829	0.888	0.446	1.000	0.803	0.442	0.427
Cream, sweet or sour cream, for food or beverages	0.990	0.412	0.720	0.726	0.701	0.923	0.720	0.561	0.503
Other animal fats	<0.001*	0.732	0.902	1.000	0.812	0.985	1.000	1.000	0.761
Mayonnaise and dressings, i. .e., salad dressings-all types	0.127	1.000	0.912	1.000	0.808	1.000	1.000	1.000	1.000
Nuts and grains									
Nuts	0.771	0.389	0.787	0.994	0.753	0.549	0.366	0.681	0.786
Grains	0.014*	0.323	0.712	0.891	0.732	1.000	0.868	0.850	0.813
Sweets and snack									
Sugar to sweeten beverages	0.009*	0.256	0.667	0.978	0.720	0.947	1.000	1.000	1.000
Honey to sweeten food and beverages	0.968	0.953	0.893	0.961	0.826	1.000	1.000	1.000	1.000
Chocolate, chocolate candies, and candy bars	0.775	0.357	0.618	0.808	0.447	1.000	1.000	1.000	0.984
Non-chocolate candies	0.972	0.410	0.760	0.621	0.455	0.793	0.896	1.000	0.904
Biscuits and cakes	0.222	0.244	0.580	0.817	0.437	1.000	0.994	1.000	0.997
Ice cream and pudding	0.843	0.952	0.909	0.819	0.545	1.000	1.000	1.000	0.785
Salty snacks	0.260	0.706	0.681	0.736	0.586	1.000	0.919	0.768	0.645
Soft drinks									
Fruit juices and fruit nectars	<0.001*	0.376	0.603	0.695	0.465	1.000	1.000	0.995	1.000
Vegetable juices and vegetable-fruit ones	0.333	0.602	0.807	0.897	0.819	0.989	1.000	1.000	0.982
Energy drinks	0.011*	0.398	0.821	1.000	0.727	1.000	1.000	0.812	0.892
Sweetened sodas such as Fanta, Coca-Cola, Mirinda, Sprite	<0.001*	0.570	0.702	0.659	0.936	1.000	1.000	1.000	1.000
Alcoholic beverages									
Beer	<0.001*	0.981	0.688	0.719	0.711	0.866	0.468	0.366	0.671

Wine and drinks	0.013*	0.534	0.651	0.714	0.904	1.000	1.000	1.000	0.754
Vodka and spirits	<0.001*	0.163	0.061	0.001*	0.610	1.000	0.699	0.632	0.537

*$p < 0.05$ *p*-adj1 - FDR-adjusted p-values for comparisons of food consumption frequency between male and female police officers (original results are presented in Table 2

p-adj2 - FDR-adjusted p-values for correlations between food consumption frequency and BMI among male police officers (original results are presented in Table S1)

p-adj3 - FDR-adjusted p-values for correlations between food consumption frequency and FMI among male police officers (original results are presented in Table S1)

p-adj4 - FDR-adjusted p-values for correlations between food consumption frequency and FAT% among male police officers (original results are presented in Table S1)

p-adj5 - FDR-adjusted p-values for correlations between food consumption frequency and V FAT L among male police officers (original results are presented in Table S1)

p-adj6 - FDR-adjusted p-values for correlations between food consumption frequency and BMI among female police officers (original results are presented in Table S1)

p-adj7 - FDR-adjusted p-values for correlations between food consumption frequency and FMI among female police officers (original results are presented in Table S1)

p-adj8 - FDR-adjusted p-values for correlations between food consumption frequency and FAT% among female police officers (original results are presented in Table S1)

p-adj9 - FDR-adjusted p-values for correlations between food consumption frequency and V FAT L among female police officers (original results are presented in Table S1)
